# Editorial: New discoveries on calcium handling in cardiovascular pathology

**DOI:** 10.3389/fcvm.2024.1461311

**Published:** 2024-07-17

**Authors:** Andreas Rinne, Jens Kockskämper, Florentina Pluteanu

**Affiliations:** ^1^ Department of Biophysics and Cellular Biotechnology, University of Medicine and Pharmacy “Carol Davila” Bucharest, Bucharest, Romania; ^2^ Institute of Pharmacology and Clinical Pharmacy, Biochemical and Pharmaceutical Center (BPC) Marburg, University of Marburg, Marburg, Germany; ^3^ Department of Anatomy, Animal Physiology and Biophysics, Faculty of Biology, University of Bucharest, Bucharest, Romania

**Keywords:** reactive oxygen species, cardiac Ca^2+^ handling proteins, mechanotransduction, Bruton tyrosine kinase (BTK) inhibitor, protein phosphatase 2A (PP2A), mitochondria-associated proteins (MAM)

**Editorial on the Research Topic** New discoveries on calcium handling in cardiovascular pathology

Understanding how cardiac Ca^2+^ cycling is altered during disease is fundamental for the development of novel therapeutical strategies to treat myocardial dysfunction. Whereas the functions of classical Ca^2+^ handling proteins involved in cardiac excitation-contraction coupling (ECC) are well characterized (1, 2), there are secondary regulatory processes of cardiac Ca^2+^ handling that are less-well understood. This research topic focuses on novel regulatory signaling pathways that have an impact on cardiac myocyte function and contractility.

The atria are subject to atrial fibrillation (AF), the most frequent cardiac arrhythmia diagnosed in human (3). AF can originate in both atrial chambers and induces severe remodeling of cardiac tissue, which impairs atrial function and further increases the prevalence of AF. Butova et al. analyzed the contractile function of atrial myocytes (AMs) from left and right atria in a rat animal model for paroxysmal AF. The study shows that AF-induced production of reactive oxygen species (ROS) and downregulation of contractile proteins were not only chamber-specific, but dependent on the preload AMs were exposed to. At higher preload, left AMs were more sensitive to AF-induced damage and showed more efficient remodeling, accompanied by a decrease in contractile function, than right AMs. Thus, hemodynamic load of myocytes affects AF, which represents a novel aspect on chamber-specific differences in cardiac pathologies.

While the molecular mechanisms underlying myocyte contraction are well understood, little is known how myocytes use stretch as a feedback mechanism to control their length. A review by Herrera-Pérez and Lamas highlights the function of TWIK-related K^+^ channels (TREK) as mechano-transducers in the cardiovascular system. By mediating K^+^ efflux as a function of membrane stretch, TREK channels contribute to repolarization of the myocyte membrane potential at the end of systole, where myocyte contraction and stretch have reached a maximum. Furthermore, reduced TREK activity during ischemia-reperfusion injuries serves as an electrical substrate for cardiac arrhythmias. In addition, TREK channels of vascular endothelial cells fine-tune NO release in response to shear stress. Therefore, mechano-transduction by TREK channels regulates important physiological parameters of the cardiovascular system, such as cardiac action potential (AP) duration, and metabolic control of blood hemodynamics.

The drug Ibrutinib is a Bruton tyrosine kinase (BTK) inhibitor that is frequently used to treat patients with leukemia. Adverse effects of BTK inhibitors affecting the cardiovascular system include hypertension, AF and ventricular arrhythmias. Tarnowski et al. present a molecular mechanism of how Ibrutinib impairs cardiac Ca^2+^ handling. In damaged ventricular tissue, endogenous insulin-like growth factor 1 (IGF-1) can improve cardiac contraction by enhancing expression levels and activities of sarco-endoplasmic Ca^2+^ ATPase (SERCA) or L-type Ca^2+^ channels. The authors showed that Ibrutinib treatment of myocytes prevented the IGF-1-mediated increase in activities of both Ca^2+^ handling proteins. This abolished the positive inotropic effect that IGF-1 had in normal ventricular myocytes, demonstrating a molecular mechanism for adverse drug effects on the heart.

Protein phosphatase 2A (PP2A) is a regulatory protein that controls cardiac contractility at many different levels by regulating the phosphorylation status of contractile proteins, Ca^2+^ release channels and Ca^2+^ removal proteins, such as the Na^+^/Ca^2+^- exchanger (NCX) and SERCA. PP2A itself is controlled by several regulatory subunits and changes in PP2A activity have been described for multiple cardiac diseases (4). Herting et al. characterized the function of PR72, a regulatory subunit of PP2A, which is upregulated in human HF. By using a transgenic mouse model over-expressing PR72, the authors showed that abundant PR72 caused an increase in intracellular Ca^2+^ transient amplitude, which translated into hypercontractility of ventricular myocytes and increased ventricular contractile force. Facilitation of Ca^2+^ release was attributed to a sensitization of SR Ca^2+^ release channels, enhanced SERCA activity and a downregulation of NCX. This mechanism was interpreted as a putative endogenous mechanism to counteract the reduced contractility in failing myocardium and highlights the functional impact of regulatory proteins on cardiac Ca^2+^ handling.

A review by Lu et al. focuses on mitochondria-associated membranes (MAM) that form contact sites between the sarcoplasmic reticulum (SR) and mitochondria and which regulate Ca^2+^ exchange between both organelles. MAM proteins couple inositol trisphosphate receptors (IP_3_R) or ryanodine receptors type 2 (RyR2) of the SR to mitochondria and regulate mitochondrial Ca^2+^ content. For some diseases, such as diabetic cardiomyopathy, SR-to-mitochondria Ca^2+^ coupling is enhanced, causing mitochondrial Ca^2+^ overload and apoptosis, whereas in other pathologies, such as heart failure (HF), the contact sites are disrupted, leading to metabolic dysfunction. Finally, pathology-related changes in expression levels of MAM are discussed, which are implicated in cardiac dysfunction observed during HF or ischemia-reperfusion injuries.

Excessive production of ROS is a hallmark of the failing myocardium. ROS cause cardiac dysfunction by altering the function of ion channels and Ca^2+^ handling proteins involved in ECC. Currents carried by voltage-activated Na^+^ channels, such as the late Na^+^ current (I_Na,L_), are involved in controlling cardiac excitability and repolarization. I_Na,L_ activity is facilitated by protein kinase A (PKA), which is sensitive to ROS, and both, enhanced Na^+^ influx and enhanced ROS production induce arrythmias during HF (5). A study by Gissibl et al. demonstrates that pharmacological activation of I_Na,L_ alone was sufficient to increase cardiac Ca^2+^ transients and induce ventricular arrythmias, with negligible contribution of the ROS-PKA signaling axis. Instead, increased Na^+^ influx affected the electrogenic activity of NCX, which caused arrythmias. Of note, I_Na,L_ activity resulted in enhanced ROS production in ventricular myocytes by a yet unknown mechanism, which could further impair ventricular contractility via effects secondary to Na^+^ influx.

In conclusion, the articles presented above highlight how cardiac Ca^2+^ handling is regulated by novel signaling pathways, such as stretch-activated channels, auxiliary proteins of cardiac enzymes, ROS or proteins associated with MAM. Because signaling of those novel pathways is altered in cardiac pathologies, any knowledge about their function may aid in developing novel therapeutic strategies to treat cardiac dysfunctions.
